# Reliability Assessment of Metal Quantification in Formalin-fixed, Paraffin-embedded Human Liver Tissue for Studying Liver Diseases

**DOI:** 10.21203/rs.3.rs-9878473/v1

**Published:** 2026-06-02

**Authors:** Nivisa Vakeesan, Jason Xu, Jamie Roberman, Li Tao, Yang Jiang, Amanda Vitale, Andrea E. Cassidy-Bushrow, Lu Cai, Wanqing Liu

**Affiliations:** 1 Department of Pharmaceutical Sciences, Eugene Applebaum College of Pharmacy and Health Sciences, Wayne State University, Detroit, MI, USA;; 2 Department of Pediatrics, Pediatric Research Institute, University of Louisville, KY, USA;; 3 Department of Public Health Sciences, Henry Ford Health, Detroit, MI, USA;; 4 Henry Ford Health + Michigan State University Health Sciences, Detroit, MI, USA;; 5 Department of Epidemiology & Biostatistics, Michigan State University College of Human Medicine, East Lansing, MI, USA;; 6 Department of Pediatrics and Human Development, College of Human Medicine, Michigan State University, East Lansing, MI, USA;; 7 Department of Pathology, Henry Ford Hospital, Detroit, MI, USA;; 8 Department of Pharmacology, School of Medicine, Wayne State University, Detroit, MI, USA; 9 Department of Physiology, School of Medicine, Wayne State University, Detroit, MI, USA

**Keywords:** metal comparison, human liver tissue, formalin-fixed paraffin-embedded (FFPE), metallomics frozen samples

## Abstract

Formalin-fixed paraffin-embedded (FFPE) human tissues are broadly available for retrospective studies. Whether the metal levels and their interindividual variability are preserved in FFPE samples remains unclear. We aim to compare metal levels and examine their variability between paired frozen and FFPE human liver tissues. The content of 25 metals in 30 pairs of human liver tissues was quantified using Inductively Coupled Plasma Quadrupole Mass Spectrometer and compared. Three metals, cadmium (Cd), iron (Fe), and zinc (Zn) exhibited linear and rank-based correlation (Pearson coefficient >0.5). Three metals (lead (Pb), aluminum (Al), and selenium (Se)) were linearly correlated but showed weak to moderate rank-based correlation (Pb and Al Kendall’s Tau <0.3 and Se Kendall’s Tau was 0.42). Our study suggests that FFPE liver tissue can be utilized to study the impact of at least three metals (cadmium, iron, zinc) on liver diseases. Selenium had a moderate relationship and is worth investigating further.

## Introduction:

Exposure to various metals is a risk factor for chronic liver disease, e.g. steatotic liver disease (SLD), steatohepatitis, fibrosis, cirrhosis, and liver cancer. Many metals accumulate in human liver tissue, which could have detrimental or certain beneficial impacts, with many trace metals playing key roles in regular cellular and biochemical functions [1–17].

As the most common chronic liver disease, metabolic-dysfunction associated steatotic liver disease (MASLD) is manifested as a spectrum of liver histology ranging from simple steatosis to a more advanced stage, steatohepatitis (MASH), characterized by hepatic cell death, hepatocyte ballooning, lobular inflammation and fibrosis [18–22]. The pathogenesis of MASLD and MASH typically involves interaction between genetic make-up and environmental exposures [23–39]. Humans (and thus, the human liver) are exposed to various metals and trace elements in daily life; understanding how metals interact with the human genome in the development of various liver histology is critical to elucidating the disease mechanisms as well for developing new strategies for disease prevention and treatment. Indeed, numerous epidemiological studies have indicated associations between metal exposures and MASLD/MASH [40–58].

However, most published studies in humans, e.g. epidemiological analyses in this field focused on metal levels in the environment or in extrahepatic biospecimens, e.g. blood, urine, hair or nails [41, 43–46, 49, 50, 52, 53, 55, 57, 58]. The direct correlation between the hepatic level of metals and the spectrum of liver histopathology underlying MASLD and MASH in human populations remains incompletely explored. This is a critical knowledge gap since metal levels in body fluids or extrahepatic tissue may not reflect that of the liver tissue [2, 10, 56]. Currently, few studies have quantitatively explored the associations between biopsy-based hepatic levels of certain metals and non-cancerous liver perturbations in humans, which are largely limited by small sample size, targeted measurements of specific metals, narrow focus on high-exposure contexts, etc. [56, 59, 60]. This is at least in part attributed to the limited access to human liver biopsy samples.

Formalin-fixed paraffin-embedded (FFPE) human tissue biopsies are the most commonly available samples for retrospective studies. However, whether the metal levels as well as their interindividual variability are preserved during sample fixation and preparation remains unclear. The few studies published thus far focusing on comparing quantitative metal data between paired fresh/frozen and FFPE liver tissue samples are limited by being non-human study, small sample size, few metals, etc. [61–70].

We aim in this study to compare levels of 25 metals and trace elements and examine the preservation of their inter-individual variability between paired frozen and FFPE human liver tissues. Our goal is to assess which metals and elements are suitable for toxicological, biochemical or nutritional analysis in human FFPE samples.

## Results

In the present study, we quantified the levels of 25 metals and trace elements within paired frozen and FFPE human liver tissues. Our frozen human non-cancerous liver tissues were procured during autopsy or biopsy and were sent to one of the two tissue banks from which we received the tissues (the Cooperative Human Tissue Network (CHTN) or National Institute of Child Health and Human Development (NICHD) Brain and Tissue Bank for Developmental Disorders). Demographic information is presented in the methods section and supplementary tables (supplementary Table S1). We present the overall distributions, statistical differences, and correlations between the paired tissues in the subsections below.

### Distribution of Metal Content

The content of 25 metals from 30 unique non-cancerous liver tissue samples (each with a paired FFPE and fresh frozen sample) were quantified. Among all metals, 17 are detectable in all samples. Among all frozen tissues, only one metal (Uranium, U) was non-detectable in more than 10 samples. For FFPE samples, more metals had non-detectable levels in over 10 samples (beryllium, Be; thorium, Th; and thallium, Tl). In general, many metals demonstrated considerable level of inter-individual variability in frozen tissues. Some extreme examples include lead (Pb), arsenic (As), chromium (Cr), and copper (Cu) (coefficient of variation or CV>200%) (Table 1). Notably, both previously established hepatoprotective metals e.g. selenium (Se) and zinc (Zn) as well as hepatotoxic metals such as Cd and Pb are all detectable among both frozen and FFPE samples with inter-individual difference in their content ranging from several to a few thousand folds (Table 1). Boxplots of the data are presented in Figure S1.

Relationship between metal data and demographic data (age, race and sex) was assessed only in frozen samples as frozen tissues provides a “gold standard” representative metal content (Table S2). When comparing between “White/Caucasian” and “Non-White” races, only manganese (Mn), showed significant difference between the two groups (nominal, unadjusted p = 0.03). When comparing between biologically female or male samples there were no statistically significant differences between the two biological sexes. The correlation between metals and age was assessed with Kendall’s Tau, no metals showed correlation (Table S2).

Paired sign test demonstrated that the metal data generally shows a significantly higher level among the FFPE sample compared to their paired frozen sample. This is evident by the statistical significance (p < 0.05) for most metals (Table 2). The only metals without a significant difference between the frozen and FFPE concentrations are beryllium (Be), Mg, manganese (Mn), cobalt (Co), and thallium (Tl). Overall, we find that FFPE preservation changes the absolute tissue content of metals.

### Correlation Metrics between Frozen and FFPE Preservations

**Figure 1:**
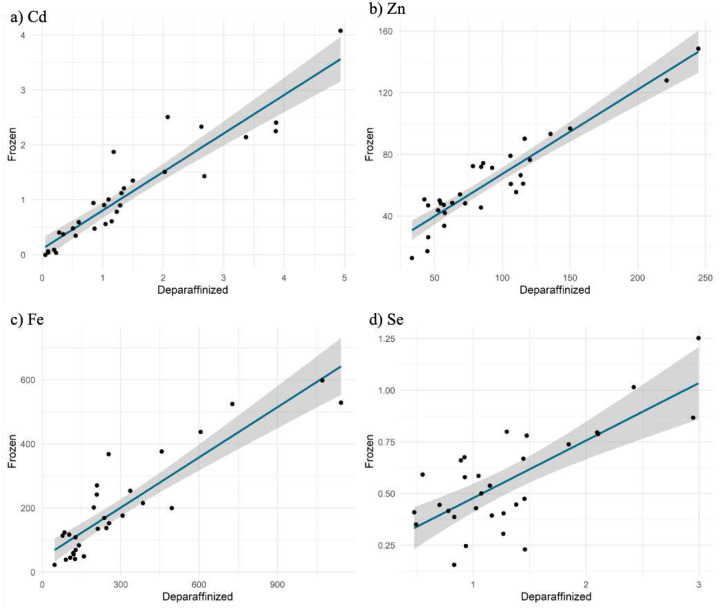
Correlation plots of the 4 metals (a. Cd, b. Fe, c. Se, and d. Zn) with the strongest Pearson correlations (in alphabetical order). 95% confidence interval is shaded in grey. X and Y axes unites are in ng/mm^3^ for the respective preservation method.

To explore whether the interindividual variability of the metal levels is preserved after FFPE preparation, we conducted correlation analysis in the metal levels between frozen and their paired FFPE samples. Table 3 shows correlation metric estimates based on the collected data and the 95% confidence interval (CI) resulting from the parametric bootstrap. Zn, Cd, and Fe had the three strongest estimates for each of the correlation measures, with Kendall’s Tau as 0.69, 0.82, and 0.67, respectively. Se had a moderate correlation between frozen and FFPE concentrations (Kendall’s Tau = 0.42). To assess whether there is a linear relationship in the metal levels between the two fixations for these four metals, we also assessed the Pearson correlation. These four metals (Zn, Cd, Fe, and Se) demonstrated strong Pearson correlation in their levels between frozen and FFPE samples, with a coefficient of 0.93, 0.93, 0.90, and 0.74, respectively. Results for these four metals are visualized in Figure 1. Summary forest plots for Kendall’s Tau and Pearson correlation are in supplement Figure S2. Individual plots for all metals can be found in the supplement Figure S3.

## Discussion:

In this study, we aimed to investigate the relationship of quantity of metals in human liver tissues between paired frozen and FFPE preservation procedures. Our primary goal is to identify which metals among FFPE liver tissue still preserve their levels and/or inter-individual variability as they are in frozen format. Our data suggest that Zn, Cd, Fe, and possibly Se are suitable for large-scale association studies on various liver disease among human populations by leveraging the broadly archived FFPE liver biopsies. As an exploratory pilot study, these results should be primarily utilized for hypothesis-generation and not confirmation as we did not perform power analysis or adjustment for multiple comparisons.

Our analysis revealed that concentrations (ng/mm^3^ tissue) of the FFPE samples tended to be greater than those of the frozen samples for most metals. We hypothesize that this is due to the shrinkage of tissue during the fixation process. Importantly, our study shows the level of four metals (Zn, Cd, Fe, and Se) were moderately to strongly correlated between frozen and FFPE samples. The reason underlying this high correlation in the level of these metals between the paired samples could be due to their chemical and biochemical properties. Chemically, these all have a charge of 2+ (Fe is sometimes present as 3+). Both Zn and Cd are known to bind metallothionein proteins [71]. Zn is an antioxidant involved in various antioxidative and detoxification processes within the liver. There are over 3,000 proteins in which zinc is incorporated across various protein classes [72, 73] which could provide as protection from washout during preservation. Cd is a toxic heavy metal that is known to cause toxicity by replacing Zn and Se in their respective processes, especially selenoproteins and metalloproteins [74, 75]. This binding is generally irreversible, and the half-life of Cd is generally decades long, leading to chronic issues within the liver [74, 75]. Fe is generally stored in the liver, primarily through ferritin [76–78]. Therefore, the complex binding with various proteins could be the primary reason leading to minimal leaching during the FFPE preservation process.

Pb and Al had strong Pearson correlation (coefficient = 0.68 and 0.56, respectively) between the frozen and FFPE samples. However, under the Kendall correlation analysis this relationship is no longer significant (Kendall’s Tau = 0.26, and 0.16, respectively), suggesting a poor concordance in general between the two tissue preparations. Upon analysis of the correlation plots (Figure S3) we find that the moderate Pearson correlation, but weak Kendall’s Tau is likely due to outlier and clustering of the concentration values. For aluminum, the frozen values have a narrower range than the FFPE concentrations. Meanwhile, in Pb, most of the concentrations have a low value in both frozen and FFPE, but potential outliers are skewing the linear correlation. It is likely that with a larger sample size these two metals can be cautiously studied with certain corrections and larger sample sizes.

There are several limitations in our study. One potential problem of our study is the normalization method. Since we were unable to dry tissues prior to digestion for ICP-MS as done in previous comparative analyses, we had to normalize the tissues utilizing other methods [65, 70]. The frozen tissues were easy to weigh given the larger size. However, deparaffinized FFPE tissues are miniscule, and an extremely sensitive scale was not available. Thus, we utilized computational methods to estimate the volume of the FFPE tissues and then estimated weight by published normal liver density (1 kg/L) [79–82]. However, it is also known that livers with increased fat content have slightly decreased densities (down to 0.90 kg/L) while livers with fibrosis have slightly increased densities (up to 1.10 kg/L). This may have slightly skewed our weight estimates. While our FFPE tissues were imaged and thus we could utilize computational methods to estimate the appropriate density, this has not been done previously and the estimates on how to adjust the density are debatable. Due to this, the normal liver density in the literature was utilized for normalization, which could lead to errors in estimating the metal content in FFPE samples.

Second, although our sample size is bigger than most published similar studies as summarized in Introduction, to thoroughly conclude which metals have a strong relationship between preservation methods, a larger sample size would still be required. In addition, it may not be valid to simply assume that the correlation of the metal levels among the paired samples is linear, and thus the Kendall’s Tau results are more appropriate.

In this study, we found that out of the 25 metals analyzed, most of the metals are detectable among the majority of samples regardless of preservation type (frozen vs FFPE). Metal levels tend to show significant differences after FFPE preservation in comparison to the original flash frozen tissues. We found that Zn, Cd, and Fe present strong linear relationships between frozen and FFPE tissue and can be utilized for future correlative studies utilizing archived liver tissues. Se, a hepatoprotective element, may also be considered for future correlative studies as it had moderate Pearson correlation and moderate Kendall’s Tau. Al, and Pb need to be investigated in larger datasets to assess the nature of the relationship as they presented with high linear correlation but weak rank-based correlation. Overall, this study indicates that while many metals are not consistent within liver tissues after the formalin-fixed and paraffin-embedding process, some metals show strong enough linear relationships to be investigated by ICP-MS.

## Materials and Methods

### Sample Collection

Frozen non-cancerous liver tissues were either from surgical biopsies or autopsies from the Cooperative Human Tissue Network (CHTN) or National Institute of Child Health and Human Development (NICHD) Brain and Tissue Bank for Developmental Disorders. All samples were de-identified prior to receipt. Both original sample collection (IRB 2018–42) and the current study (IRB-24-04-6777) using these samples were approved by the Institutional Review Board (IRB) of the Wayne State University. The demographics are presented in Table S1.

### Fresh vs. Formalin Fixation

Randomly chosen samples were cut with a carbon blade (Electron Microscopy Sciences, Cat# 71964, Hatfield, PA, USA) to prevent metal contamination. All samples were cut on the same day utilizing a new blade. One piece of tissue was weighed and placed in a 1.7 mL Eppendorf tube and kept in −80 until shipping to Dr. Cai’s lab at the University of Louisville (Louisville, KY, USA) under dry ice. An adjacent piece of tissue was cut and placed in a cassette for formalin fixation. Immediately after being placed on the cassette, the samples were placed in 10% formalin for fixation. After 24 hours, tissues were placed in ethanol for 12 hours. Tissues were subsequently processed utilizing the Tissue Tek VIP (Miles Scientific, Newark, DE, USA) and embedded in paraffin. FFPE tissue blocks were stored at room temperature until sectioning. 10 μM sections of tissue were deparaffinized and imaged prior to placing in a 1.7 mL Eppendorf tube. Frozen and FFPE tissues were digested and metals were quantified by using Inductively Coupled Plasma Quadrupole Mass Spectrometer (ICP-MS) at Dr. Cai’s lab (See below). The purpose of imaging the tissue section is to calculate the tissue volume for normalization of metals. The imaging was conducted by scanning each section in the Odyssey M (Licor, Lincoln, NE, USA) utilizing the slide image setting. Five samples were randomly chosen to be duplicated under this procedure for both frozen and FFPE tissues.

### Tissue Digestion

Liver samples (15–70 mg) and deparaffinized sections as prepared above were placed in 1.7 mL Eppendorf tubes and digested with addition of 500 μl 70% nitric acid (Fisher Scientific trace metal grade, Cat# A509P500, Waltham, MA, USA). To ensure efficient digestion (until solution becomes clear without residues), the samples containing nitric acid were gently shacked at 65°C for 4 hours. Then the samples were diluted with 6.5 ml deionized water (Thermo Fisher Scientific, GenPure Pro, Waltham, MA, USA) into final 5% acid sample solutions for ICP-MS assay. Three reagent samples for blank controls were also similarly treated at the same time with tissues.

### Tissue Sample ICP-MS Analysis

To measure the metal content in tissue samples with ICP-MS, the Agilent 7800 ICP-MS (Agilent Technologies, Japan) was optimized by performance check with 1ppb tuning solution. The assay program was auto tuned by 10 ppb tuning solution (Agilent Cat#5188–6564, Santa Clara, CA, USA). Sample solutions were introduced automatically by the SPS 4 auto-sampler. Twenty-five metals calibration standard, purchased from Inorganic Ventures (Cat# IV-STOCK-50, Christianburg, VA, USA), were included with serial metal standard dilutions in the same acid matrix of samples. Internal standard was purchased from Agilent (Cat#5188–6525, Santa Clara, CA, USA) and lithium (Li), scandium (Sc), germanium (Ge), rhodium (Rh), indium (In), terbium (Tb), and bismuth (Bi) were using as internal standard for metal recovery rate. Assay program was run by Agilent MassHunter software with He mode and each sample was read 3 times for final mean value. Standard solutions with 20ng/ml (trace metals) and 2000 ng/ml (sodium (Na), potassium (K), calcium (Ca), magnesium (Mg), and iron (Fe)) were used for quality control. Detailed parameters for ICP-MS setup are included in Table S3.

### Data Preparation and Calculation of Metal Concentration

Metal concentrations were multiplied by the respective assay volume to find the total amount of metal in each sample. This total amount was then divided by the sample’s volume to have a normalized value of ng/mm^3^. Prior to ICP-MS analysis, FFPE samples were imaged on Odyssey M (Licor, Lincoln, NE, USA) utilizing the slide image setting then scales were added from the software of the instrument. The images were then taken to ImageJ [83, 84] for area quantification. All sections were cut at 10-micron thickness, and this was utilized to obtain the estimated volume of sections. For frozen tissues, since volume was not easily measured without potential contamination, a normalization factor of 1 kg/L was utilized. This value comes from the density measured in various studies [79–82].

### Statistical Analysis

Metals with at least 10 (33%) samples with nondetectable frozen or FFPE concentrations were excluded from the correlation analysis. Analysis was performed in R utilizing base functions and *dplyr* package for data manipulation for both raw and natural log transformed data. Normality was assessed visually and with Shapiro-Wilk tests. Based on these assessments, we concluded that most metals were not normally distributed and opted to use non-parametric analysis methods. Therefore, non-parametric analyses based on non-transformed raw values were primarily applied to the subsequent analyses.

Associations between metal data (frozen samples only) and demographics [age, sex, race (white vs non-white)] were performed using Kendall’s Tau correlation (Age) and Mann-Whitney U test (sex and race). Comparison of metal concentrations between FFPE and frozen samples was conducted with paired Sign test. Samples with paired measurements in both sample types were included for analysis. To assess the correlation in the metal levels between the FFPE and frozen samples, Kendall rank correlation coefficient (Tau) was estimated. However, given the major goal of this study is to assess whether there is a linear relationship in the metal levels between the frozen and paired FFPE samples, we also conducted Pearson correlation using log transformed data. To quantify the uncertainty in these measures, we present each estimate with an associated 95% confidence interval (CI), calculated using Bias-Corrected and Accelerated (BCa) bootstrap methodology utilizing the confintr R package[85]. Given that the correlation is between paired samples, no additional adjustment for co-variables e.g. demographic data was applied. For all statistical tests, significance of statistics was defined as p<0.05. Due to this being an exploratory pilot study, we did not adjust for multiple comparisons. Analysis is performed in R (R Core Team, 2024).

## Supplementary Material

Supplementary Files

This is a list of supplementary files associated with this preprint. Click to download.

- npjsuppledata51326.docx

## Figures and Tables

**Table 1:** Descriptive statistics (mean (Mn), median (Md), standard deviation (SD), minimum (Min.), first quartile (1st Qu), third quartile (3rd Qu.), maximum (Max.), skewness, kurtosis, and coefficient of variation (CV) of the 25 metals quantified across the frozen and FFPE samples utilized in this study. Number of zeroes (No. of zeroes) denotes how many samples for the respective type (frozen or FFPE) and metal were below quantifiable levels via ICP-MS.

Metal	Method	No. of Zeros (n)	Mn	Md	SD	Min	1st Qu.	3rd Qu.	Max	Skewness	Kurtosis	CV (%)
Ag	Frozen	0	0.01	0.00	0.01	0.00	0.00	0.01	0.03	3.02	13.40	112.84
	FFPE	0	0.03	0.03	0.01	0.01	0.02	0.04	0.06	0.40	2.29	44.70
Al	Frozen	0	0.91	0.60	1.50	0.12	0.42	0.81	8.66	4.82	25.47	164.41
	FFPE	0	17.27	14.08	7.98	6.40	11.97	21.47	42.08	1.26	4.60	46.22
As	Frozen	0	0.01	0.00	0.03	0.00	0.00	0.01	0.15	4.94	26.20	254.39
	FFPE	10	0.02	0.02	0.02	0.00	0.00	0.03	0.08	0.94	2.70	106.93
Ba	Frozen	0	0.03	0.02	0.02	0.00	0.01	0.04	0.08	0.77	2.36	88.69
	FFPE	0	0.55	0.49	0.32	0.07	0.33	0.63	1.53	1.37	4.83	58.34
Be	Frozen	7	0.00	0.00	0.00	0.00	0.00	0.00	0.00	1.97	8.94	95.34
	FFPE	12	0.04	0.02	0.05	0.00	0.00	0.06	0.21	1.82	6.92	129.13
Ca	Frozen	0	50.29	44.75	23.25	20.16	32.13	61.20	120.19	1.33	4.73	46.22
	FFPE	0	626.13	386.79	435.91	216.48	316.49	769.02	2020.37	1.44	4.70	69.62
Cd	Frozen	0	1.10	0.90	0.95	0.00	0.42	1.49	4.08	1.18	4.30	86.98
	FFPE	0	1.41	1.12	1.26	0.05	0.52	1.89	4.93	1.22	3.73	89.56
Co	Frozen	0	0.04	0.04	0.02	0.00	0.03	0.05	0.09	−0.01	3.58	40.76
	FFPE	0	0.05	0.05	0.02	0.01	0.04	0.06	0.11	0.38	2.52	43.50
Cr	Frozen	0	0.11	0.03	0.25	0.01	0.02	0.09	1.28	3.77	17.52	218.81
	FFPE	0	1.22	0.87	1.32	0.26	0.58	1.07	6.26	2.83	10.50	108.24
Cu	Frozen	0	11.55	6.30	24.50	1.17	4.75	9.71	139.86	5.03	26.86	212.15
	FFPE	0	53.10	16.88	78.65	8.91	12.56	26.41	266.07	1.76	4.51	148.14
Fe	Frozen	0	197.02	144.81	159.54	22.65	72.79	250.52	597.93	1.12	3.26	80.97
	FFPE	0	292.17	208.38	274.14	46.71	120.77	329.03	1142.86	1.92	6.05	93.83
K	Frozen	0	2353.23	2376.75	579.18	890.58	2102.51	2828.47	3257.18	−0.70	2.85	24.61
	FFPE	4	38.95	30.09	40.03	0.00	13.84	42.61	163.48	1.94	6.70	102.78
Mg	Frozen	0	156.51	159.26	39.10	60.55	141.82	181.19	224.55	−0.75	3.44	24.98
	FFPE	0	143.31	134.22	80.43	35.58	80.67	211.31	309.83	0.41	2.00	56.13
Mn	Frozen	0	1.41	1.34	0.61	0.23	1.07	1.82	2.53	−0.03	2.50	43.07
	FFPE	0	1.70	1.51	0.89	0.50	1.09	2.12	4.42	1.43	5.07	52.27
Mo	Frozen	0	0.68	0.63	0.36	0.04	0.47	0.94	1.41	0.31	2.30	53.29
	FFPE	0	1.01	0.89	0.68	0.28	0.64	1.13	3.32	1.84	6.36	67.33
Na	Frozen	0	1198.02	1176.65	406.35	561.25	930.85	1406.05	2071.61	0.53	2.49	33.92
	FFPE	0	456.04	375.51	376.94	36.38	101.13	764.99	1234.12	0.48	1.95	82.66
Ni	Frozen	0	0.03	0.01	0.06	0.00	0.01	0.02	0.30	3.72	16.70	196.15
	FFPE	0	8.63	0.54	38.00	0.08	0.14	0.96	207.78	5.05	26.92	440.49
Pb	Frozen	0	0.07	0.02	0.22	0.00	0.01	0.05	1.25	5.07	27.16	304.58
	FFPE	0	0.38	0.13	0.69	0.05	0.10	0.25	2.87	2.81	9.47	182.84
Sb	Frozen	0	0.00	0.00	0.00	0.00	0.00	0.00	0.02	3.22	13.45	119.49
	FFPE	1	0.03	0.02	0.04	0.00	0.02	0.04	0.23	3.85	19.04	125.28
Se	Frozen	0	0.56	0.52	0.24	0.16	0.41	0.72	1.25	0.74	3.51	43.18
	FFPE	0	1.31	1.16	0.65	0.48	0.90	1.46	2.99	1.18	3.84	49.77
Th	Frozen	9	0.00	0.00	0.00	0.00	0.00	0.00	0.00	3.27	15.12	151.70
	FFPE	21	0.01	0.00	0.01	0.00	0.00	0.01	0.05	2.47	8.37	209.73
Tl	Frozen	2	0.00	0.00	0.00	0.00	0.00	0.00	0.00	3.07	12.08	154.21
	FFPE	28	0.00	0.00	0.00	0.00	0.00	0.00	0.01	3.72	15.41	388.83
U	Frozen	18	0.00	0.00	0.00	0.00	0.00	0.00	0.00	1.68	5.79	151.49
	FFPE	5	0.01	0.01	0.00	0.00	0.01	0.01	0.02	−0.14	2.68	59.33
V	Frozen	0	0.01	0.00	0.01	0.00	0.00	0.01	0.05	3.15	13.27	166.92
	FFPE	6	0.02	0.02	0.02	0.00	0.01	0.02	0.08	1.57	5.47	98.15
Zn	Frozen	0	62.04	54.81	29.19	12.73	47.01	73.83	148.57	1.03	4.48	47.05
	FFPE	0	90.43	81.30	49.48	33.32	55.05	112.66	244.68	1.57	5.45	54.72

**Table 2:** Results of paired sign test comparing the FFPE samples to their respective frozen sample to examine if there is a significant change in metal levels after FFPE preservation. If a sample was below quantifiable levels for either the frozen or FFPE sample, it was removed from the analysis. If pairs were removed, then the n value in the n pairs column is less than 30. Significance (yes vs. no) is determined based on a p value less than 0.05, without adjustment for multiple comparisons.

Metal	Number of pairs	Sign test statistic value	Sign test p value
Be	9	1.13	0.26
Na	30	−4.02	**<0.001**
Mg	30	−1.46	0.14
Al	30	5.48	**<0.001**
K	24	−5.48	**<0.001**
Ca	30	5.48	**<0.001**
V	24	2.56	**0.01**
Cr	30	5.11	**<0.001**
Mn	30	1.10	0.27
Fe	30	3.29	**<0.001**
Co	30	1.83	0.07
Ni	30	5.11	**<0.001**
Cu	30	5.11	**<0.001**
Zn	30	4.75	**0.00**
As	19	2.19	**0.03**
Se	29	4.75	**<0.001**
Mo	30	2.19	**0.03**
Ag	30	5.48	**<0.001**
Cd	30	3.65	**<0.001**
Sb	29	5.11	**<0.001**
Ba	30	5.48	**<0.001**
Tl	2	−4.54	**<0.001**
Pb	30	5.48	**<0.001**
Th	9	−0.85	0.39
U	8	4.27	**<0.001**

**Table 3:** Correlation statistics for each metal between the frozen and FFPE samples from Kendall’s Tau and Pearson Correlation tests. For each test, the estimated (Est.) correlation value, 95% confidence interval (CI), width, and p value are presented. Metals with 10 or more below quantifiable level concentrations were removed from the analysis.

Metal	Kendall's Tau Est.	Kendall's Tau 95% CI	Kendall's Tau Width	Pearson Est.	Pearson 95% CI	Pearson Width
Ag	0.2	(−0.09, 0.45)	0.54	0.27	(−0.08, 0.50)	0.57
Al	0.16	(−0.15, 0.44)	0.59	0.56	(−0.25, 0.90)	1.14
Ba	−0.01	(−0.31, 0.27)	0.58	0.06	(−0.32, 0.58)	0.9
Ca	0.16	(−0.04, 0.35)	0.39	0.18	(−0.06, 0.37)	0.43
Cd	0.82	(0.70, 0.90)	0.2	0.93	(0.83, 0.97)	0.14
Co	0.18	(−0.15, 0.48)	0.63	0.15	(−0.32, 0.60)	0.92
Cr	0.16	(−0.14, 0.40)	0.54	−0.05	(−0.21, 0.13)	0.34
Cu	0.15	(−0.15, 0.39)	0.54	0.29	(−0.23, 0.86)	1.09
Fe	0.67	(0.50, 0.81)	0.31	0.9	(0.73, 0.95)	0.22
K	0.06	(−0.23, 0.36)	0.59	−0.26	(−0.71, 0.21)	0.91
Mg	0.28	(0.05, 0.50)	0.44	0.45	(0.16, 0.67)	0.51
Mn	0.4	(0.21, 0.52)	0.31	0.47	(0.23, 0.63)	0.4
Mo	0.34	(0.07, 0.56)	0.49	0.47	(0.16, 0.71)	0.55
Na	0.07	(−0.17, 0.27)	0.44	0.1	(−0.21, 0.38)	0.6
Ni	0.09	(−0.22, 0.38)	0.6	−0.03	(−0.18, 0.40)	0.58
Pb	0.26	(0.01, 0.47)	0.46	0.68	(−0.05, 1.00)	1.05
Sb	0.06	(−0.21, 0.34)	0.54	0.07	(−0.23, 0.48)	0.71
Se	0.42	(0.11, 0.64)	0.53	0.74	(0.47, 0.88)	0.41
V	0.08	(−0.19, 0.36)	0.55	0.32	(−0.19, 0.71)	0.9
Zn	0.69	(0.50, 0.83)	0.33	0.93	(0.83, 0.97)	0.14
